# Study on the Formation Mechanism of Medical and Health Organization Staff’s Emergency Preparedness Behavioral Intention: From the Perspective of Psychological Capital

**DOI:** 10.3390/ijerph18168246

**Published:** 2021-08-04

**Authors:** Huihui Wang, Jiaqing Zhao, Ying Wang, Yuxiang Hong

**Affiliations:** 1 School of Law and Public Administration, Hunan University of Science and Technology, Xiangtan 411201, China; 1020097@hnust.edu.cn; 2 School of Management, Hangzhou Dianzi University, Hangzhou 310018, China; zhaojiaqing@hdu.edu.cn; 3 Enze Hospital of Taizhou Enze Medical Center (Group), Taizhou 318050, China; wangy3524@enzemed.com

**Keywords:** psychological capital, theory of planned behavior, structural equation model, MHO staff, emergency preparedness behavior

## Abstract

Medical and Health Organization (MHO) staff’s emergency preparedness awareness and behaviors are essential variables that affect public health emergency response effectiveness. Based on the theory of psychological capital and the theory of planned behavior (TPB), this study discusses the mechanism of the psychological characteristics of MHO staff on their emergency preparedness behavioral intention (EPBI). To verify the research model, we conducted a web-based questionnaire survey among 243 MHO staff from China and analyzed the data using the structural equation modeling software, AMOS 24.0 (IBM, New York, United States). The empirical results reveal that psychological capital significantly affected cognitive processes theorized by TPB. This study suggests that the positive psychological capital of MHO staff should be developed and managed to improve their EPBI.

## 1. Introduction

With deterioration of the natural environment, changes in the international situation, and the acceleration of domestic, economic, and social reform, the frequency of various natural disasters, accident disasters, mass incidents, and public health events has shown a significant upward trend [1,2,3,4]. This has become a significant problem affecting economic development, social governance, and even national security. Globally, China is one of the countries with severe disaster risk situations; all types of accidents, hidden dangers and safety risks are prone to occur frequently and continue to evolve into social crises [5]. In particular, the large-scale spread of COVID-19 in the early stage of the pandemic has caused some shortcomings in China’s paramount epidemic prevention and control system, public health emergency management system, etc., especially in prevention and early warning, advance disposal, emergency material reserve supply, and other aspects of the lack of necessary adaptability and response [6,7]. Currently, China is in a critical period of moving from a large developing country to a modern social power; the importance and urgency of people’s livelihoods and well-being, and social and economic construction, together put forward higher requirements for the national emergency management level [8]. However, the key to excellent emergency management is to strengthen emergency preparedness, which should run through the entire process of dealing with emergencies [7].

Emergency preparedness is a sub-field of emergency management, mainly referring to the establishment and maintenance of various preparations for emergency prevention, early warning, response, and recovery. It includes emergency plan formulation, personnel training, preparation and custody of emergency supplies and equipment, drills within the emergency plan, and connection with external emergency forces. Its ultimate goal is to maintain the emergency capacity and rapid response capacity needed for emergency rescue in relation to significant accidents and ultimately reduce casualties and unnecessary losses [9]. The Medical and Health Organization (MHO) is the main institution for the treatment of diseases and wounds, and its goal is to protect and improve people’s health, including hospitals, grass-roots medical and health institutions, professional public health institutions, etc. It is undeniable that no matter what kind of disaster or accident occurs, MHO staff (e.g., doctors, nurses, hospital administrators, CDC staff, health management staff) are at the forefront of response. For example, after an emergency public health incident, the CDC will formulate effective prevention and control measures as soon as possible; the medical and health teams will also go to the disaster areas to assist in medical treatment, health prevention and psychological assistance. As the main force of the rescue effort, they are also the core force in the construction of the national emergency system, and their emergency preparedness capacity and motivation directly affect the quality of medical rescue. Therefore, strengthening the emergency preparedness of MHO staff is also an essential task of national emergency capacity construction [10]. However, currently, few studies have focused on the MHO staff’s behavior related to emergency preparedness.

In order to fill the gap in this field, we try to use the theory of planned behavior (TPB) to understand the self-driving mechanism of MHO staff’s emergency preparedness behavioral intention (EPBI). In this study, EPBI is considered to be the intention of MHO staff to prepare so as to avoid losses from emergencies. As an essential theory to explain the general decision-making process of individual rational behavior, TPB is widely used in the field of behavioral science, and has been proved to have good explanatory and predictive power of human behavior, and can help researchers understand how people change their behavior patterns [11,12,13]. TPB holds that thoughtful and planned behavior comes from behavior intention, which depends on people’s attitude towards behavior, subjective norms, and perceived behavioral control [14]. However, TPB is not an omnipotent theory, it has a strict scope of application. For example, TPB is based on the premise of completely rational people, and cannot explain well the individual behavior related to emotion and community [15,16]. Therefore, the traditional TPB model is not suitable to predict and explain all behaviors in specific areas, especially those with a wider range, higher conditions, more initiative and beyond the formal requirements of the position. In today’s increasingly tricky emergency management situation, the government calls on all MHO staff to be prepared for emergencies, and to respond in order to minimize the damage caused by the accident. These behaviors generally go beyond the job description of MHO employees, and are undertaken entirely out of personal will and have nothing to do with the formal reward system, nor the behavior required by the role. This requires MHO staff to have a broader level of competence, which requires the use a large number of resources that can motivate them to take the initiative to perform a wider range of tasks.

Therefore, we attempt to add psychological capital (PsyCap) as an antecedent factor to the TPB model in order to better understand the formation mechanism of MHO staff’s EPBI, and simultaneously stimulate their subjective initiative to participate in emergency preparedness work. The psychological capital proposed by the American management scientist Luthans et al. is regarded as a positive psychological state in the process of individual growth and development, a core psychological element beyond human capital and social capital, and a psychological resource to promote personal growth and performance improvement, including hope, optimism, resilience, and self-efficacy [17,18,19]. Among these factors, hope refers to “a positive motivational state of success based on the interaction between agents (goal-oriented vitality) and paths (plans to achieve goals)”; optimism is the characteristic of individuals who “expect things to go their way, and generally believe that good, rather than bad, things will happen to them”; resilience is “the positive psychological capacity to rebound, to ‘bounce back’ from adversity, uncertainty, conflict, failure, or even positive change, progress and increased responsibility”; self-efficacy is a role-breadth characteristic and is defined as an “employee’s perceived capability of carrying out a broader and more proactive set of work tasks that extend beyond prescribed technical requirements” [20]. The PsyCap study calls on people to turn their attention to individuals’ positive, effective, and efficient aspects, rather than focusing on correcting their problems [21]. Previous studies proved PsyCap to be a kind of psychological quality similar to the state described, and related to specific tasks, situations, and environment; it will change with time and has strong plasticity [22]. More studies support this view, suggesting that PsyCap can be developed through interventions, and influence individual action processes [23,24,25]. This makes it possible to develop the PsyCap of MHO staff as a positive way to promote their EPBI. Moreover, PsyCap is also regarded as a role-width resource, which is a further expansion and extension of the positive psychological movement in the field of active behavior research, emphasizing the broader role competence of staff members. It helps staff to participate in out-of-role behavior in a more active state. Therefore, this study believes that for the emergency rescue work with MHO staff as the backbone, the positive role that PsyCap can bring is particularly important. It is reasonable for us to explore the intermediary mechanism of TPB in the process of the influence of PsyCap on MHO staff’s emergency preparedness behavior.

## 2. Research Hypotheses and Theoretical Model

### 2.1. TPB and EPBI

In this study, attitude refers to MHO staff’s evaluation of their psychological tendency to conduct emergency preparedness. Perceived behavioral control refers to the difficulty or ease that MHO staff feel when responding to emergency preparedness. Finally, subjective norms refer to the social pressure that MHO staff feel when deciding whether to conduct emergency preparedness, primarily obtained by consulting or observing others’ behavior [14].

Firstly, TPB believes that an individual’s attitude towards behavior will affect his or her behavioral intention. In a specific time and environment, individuals can acquire a small amount of beliefs about behavior, which are the cognitive and emotional basis of attitude. Among them, individuals with positive beliefs and values about emergency-related content, knowledge, and skills tend to participate in emergency prevention and preparedness, such as emergency knowledge popularization activities and emergency training drills [26]. Those who lack such a good attitude will not continue to conduct the relevant preparatory work [27]. Many previous studies have also confirmed that MHO staff’s attitude towards behavior has a positive impact on their behavior intention [28,29,30,31,32]. Therefore, this study infers that a positive emergency attitude indicates a good EPBI. Conversely, a negative attitude reduces an individual’s EPBI. Based on the above discussion, this study proposes the following assumption.

**Hypothesis** **1** **(H1)**. *MHO staff’s attitude towards emergency preparedness behavior has a positive impact on their EPBI.*

Secondly, TPB also believes that perceived behavioral control is related to behavior intention. Perceived behavioral control also emphasizes an individual’s ability to cope with tasks or make choices to a certain extent, and this ability perception mainly comes from a sense of self-efficacy [33]. Previous studies have shown that self-efficacy is significant in improving levels of responsibility taken in an emergency and work enthusiasm of the MHO staff [34,35,36,37]. Among them, MHO staff with high self-efficacy have high expectations of themselves, are more inclined to choose challenging tasks, and will adhere to their behavior even if they encounter difficulties [38,39,40]. Conversely, individuals with low self-efficacy have low cognition and evaluation of themselves and tend to give up after being negatively affected [41]. Therefore, this study predicts that the stronger the sense of control that MHO staff perceive, the more willing they are to participate in emergency prevention and preparedness. Based on the above discussion, this study proposes the following assumption.

**Hypothesis** **2** **(H2)**. *MHO staff’s perceived behavioral control has a positive impact on their EPBI.*

Finally, an individual’s behavior is influenced or motivated by the norms observed in their environment. For example, before the disaster, if the MHO staff noticed that the people around them (superiors, colleagues, and subordinates) were making preventive preparations, they were more likely to participate actively in emergency prevention and preparedness. Conversely, subjective norms also reflect the degree of support of external factors for MHO staff’s emergency preparedness behavior to a certain extent and play a vital role in the formation of individual emergency attitudes and perceived behavioral control [42,43]. For example, when MHO staff think that not taking precautions will bring them practical benefits, and the people around them do not show any particular aversion to this behavior, they are likely to treat emergency preparedness with a negative attitude. However, the establishment of appropriate emergency safety education and training mechanisms within the organization can effectively improve MHO staff’s working skills and knowledge level and enhance the confidence and determination of internal staff to conduct emergency preparedness. Based on the above discussion, this study proposes the following assumptions:

**Hypothesis** **3** **(H3)**. *The subjective norms of MHO* *staff will have a positive impact on their EPBI.*

**Hypothesis** **4** **(H4)**. *The subjective norms of MHO* *staff will have a positive impact on their attitude towards emergency preparedness behavior.*

**Hypothesis** **5** **(H5)**. *The subjective norms of MHO* *staff will have a positive impact on their perceived behavioral control.*

### 2.2. PsyCap and TPB

Positive PsyCap reflects the following view: Firstly, regardless of whether they are facing the disaster threat, MHO staff are willing to carry out all kinds of emergency preparedness work [24]. Secondly, MHO staff with high PsyCap expect that emergency preparedness will lead to sound rather than bad results and can maintain this firm belief even if they are affected by adverse events [44]. Thirdly, MHO staff with high PsyCap have confidence in their competence to perform their roles, including emergency preparedness and responses to adverse events and potential threats [21]. Therefore, this study predicts that MHO staff with a higher PsyCap level are more willing to actively participate in emergency preparedness. Based on this, this study proposes the following assumption:

**Hypothesis** **6** **(H6)**. *MHO* *staff’s PsyCap has a positive impact on their attitude towards* *emergency preparedness* *behavior.*

In recent years, through factor analysis, researchers have found that the standard of perceived behavioral control is loaded on two factors. The former reflects the belief in self-efficacy (the individual’s judgment of their ability to perform and complete a particular behavior), and the latter reflects the belief in control (the influence of external promotional or hindering factors on the individual’s performance of a particular behavior) [45]. However, PsyCap can enhance MHO staff’s confidence that they can perform emergency preparedness work and MHO staff’s spirit that their emergency preparedness work can effectively reduce the degree of accident damage. Simultaneously, MHO staff with high PsyCap can work efficiently with a positive attitude and pay less attention to adverse problems in their work [46]. Based on the above discussion, this study proposes the following assumption:

**Hypothesis** **7** **(H7)**. *MHO* *staff’s PsyCap* *has a positive impact on their perceived behavioral control.*

As an effective way to enhance inner strength and promote individual growth, PsyCap can help MHO staff adjust to psychological and physical problems caused by inter-personal relationships and work stress and improve personal trust and satisfaction [47,48]. Therefore, MHO staff with a high PsyCap level are more willing to believe that their leaders and colleagues attach considerable importance to emergency preparedness. Thus, they also believe that it is imperative to participate in emergency preparedness. Based on the above discussion, this study proposes the following assumption:

**Hypothesis** **8** **(H8)**. *MHO* *staff’s* *PsyCap* *has a positive impact on their subjective norms.*

### 2.3. The Intermediary Role of Attitude, Perceived Behavioral Control, and Subjective Norms

With careful consideration of Hypothesis 1 to Hypothesis 8, this study puts forward the intermediary hypothesis of attitude, perceived behavioral control, and subjective norms. Based on the previous discussion, this study holds that the positive PsyCap of MHO staff will have a positive impact on their attitude towards emergency preparedness behavior, perceived behavioral control and subjective norms. And these factors will also positively affect their EPBI. Therefore, we have reason to expect that MHO staff’s attitude, perceived behavioral control and subjective norms may play an intermediary role between PsyCap and EPBI. In addition, we also believe that MHO employees’ subjective norms will promote their attitude and perceived behavioral control, and this study also proposes the intermediary role of attitude and perceived behavioral control between subjective norms and EPBI. The specific assumptions are as follows:

**Hypothesis** **9** **(H9)**. *The attitude towardsemergency preparedness behaviorof MHOstaff acts as an intermediary between PsyCap and EPBI*.

**Hypothesis** **10** **(H10)**. *The perceived behavioral control* *of MHO staff acts as an intermediary between PsyCap and EPBI.*

**Hypothesis** **11** **(H11)**. *The subjective norms of MHO* *staff act as intermediaries between PsyCap and EPBI.*

**Hypothesis** **12** **(H12)**. *The attitude towards emergency preparedness behavior of MHO staff acts as an intermediary between subjective norms and EPBI*.

**Hypothesis** **13** **(H13)**. *The perceived behavioral control of MHO staff acts as an intermediary between subjective norms and EPBI.*

**Figure 1:**
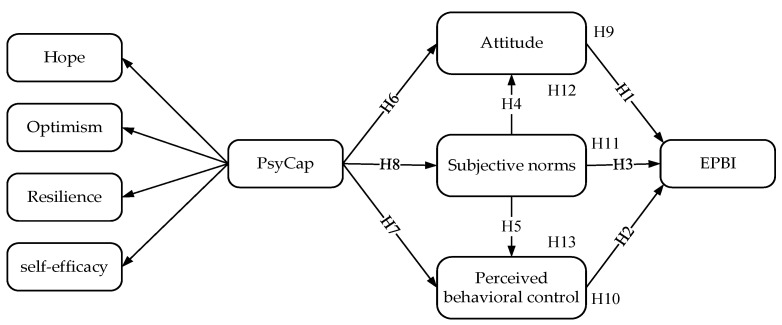
The model of influencing factors of MHO staff’s EPBI.

Figure 1 illustrates the theoretical model.

## 3. Methods

### 3.1. Study Design

We used the method of questionnaire survey to test the research model. The data sources of this study were accurate and reliable. Firstly, to avoid the systematic error caused by the deviation of the standard method, this study invited five doctoral students of related majors to revise the questionnaire repeatedly to make the question items as concise and easy to understand as possible. To avoid individual repetition, we set the questionnaire to be answered only once per IP address. Secondly, to encourage the participants to answer the questions frankly and truthfully, the online questionnaire used in this study provided complete anonymity—the researchers never knew the identity of the interviewees. Further, the survey was conducted and analyzed outside the organization—enhancing the interviewees’ perceived anonymity and actual anonymity. Finally, to ensure the diversity of data sources, this study selected a group of staff composed of staff from different MHOs in China as the research cohort. The use of group samples increases the certainty that the sampling population will accurately represent the target population, and the survey subjects are MHO staff. Therefore, the survey results are more likely to be extended to all MHO workers’ groups.

### 3.2. Measures

The measurement scales used in this study were adapted from the maturity scale proposed by previous scholars. We invited relevant professionals to translate repeatedly to avoid measurement errors caused by semantic differences. Considering that if a potential variable is measured by three or more observation variables, the estimation deviation of the model parameters is almost zero, this study retained three questions for each potential variable. The measurement items of PsyCap were adapted from the questionnaire of Luthans et al. (2007) [49]. The respondents used a 6-point Likert scale to score, ranging from “1 = strongly disagree” to “6 = strongly agree”—the higher the score, the higher the PsyCap level. The measurement items of attitude, subjective norm, and perceived behavioral control were adapted from the questionnaire of Ajzen (2006) [50]. The measurement items of EPBI were adapted from the questionnaires of Miceli et al. (2008) [51], Murphy et al. (2009) [52], Paek et al. (2010) [34] and Hong et al. (2019) [53]. The respondents scored with a 5-point Likert scale, ranging from “1 = strongly disagree” to “5 = strongly agree”. The measurement items of each variable are shown in Table 1. In addition, it was considered that the factors that affect individual emergency preparedness behavior were complex and multifaceted, including demographic characteristics, previous disaster experience, etc., [54,55,56]. Therefore, gender, age, education, occupation, department and experience were selected as control variables in this study.

### 3.3. Study Participants

Initially, the survey received responses from 289 MHO staff. After excluding incomplete answers and screening out spoiled solutions (for example, the options checked in the whole questionnaire were all the same), it was determined that the number of valid samples was 243, and the effective recovery rate was 84.1%. Among the participants, 80.7% were female, 37.4% were aged between 18 to 24, 72.1% had a Bachelor’s degree or above, and 45.7% were nurses. Additionally, 40.7% of the respondents supported Wuhan during the “COVID-19” epidemic. Table 2 shows the demographic characteristics of the participants.

### 3.4. Data Analysis

In this study, we analyzed the data in three steps. In the first stage, the reliability and validity of the measurement model were tested. In the second stage, the fitness of the structural equation model was tested. In the third stage, the structural equation model was used to test the hypotheses. SPSS 26.0 (IBM, New York, NY, USA) and AMOS 24.0 (IBM, New York, NY, USA) were used to analyze data.

## 4. Results

### 4.1. Reliability and Validity Testing

Although all the scales in this study have been recommended, their reliability and validity still need to be evaluated. Firstly, SPSS 26.0 was used to test the reliability of the questionnaire data. The results show that the Cronbach’s α coefficient of the whole questionnaire data is 0.960, and that of each variable is more significant than 0.8, indicating that the internal consistency of the scale used in this study is promising.

Secondly, we use AMOS 24.0 software to analyze the validity of the questionnaire data, including the content validity test, convergent validity test, and discriminant validity test. The results show the following: First, except for one observation variable’s standard factor load coefficient between 0.6 and 0.7, the other observation variables are all above 0.7; all of them have reached a significant level, indicating that the questionnaire has good content validity. Second, the composite reliability (CR) of each variable is more significant than 0.8, and the average variance extracted (AVE) is more significant than 0.6, indicating that the scale has good convergent validity. Third, there is no obvious distinction between the four substructures of PsyCap, but there is obvious differentiation between these four substructures and other variables, as well as other variables. Previous studies have conceptualized PsyCap into a higher-order structure [49]. Compared with the first-order structure, there are common potential factors among the substructures of the higher-order structure, and there is no need to show discriminant validity [20]. Therefore, the scale in this study has good discriminant validity. In summary, the scale developed in this study has high reliability and validity, and Table 3 and Table 4 show the specific analysis results.

### 4.2. Model Fitting

**Figure 2:**
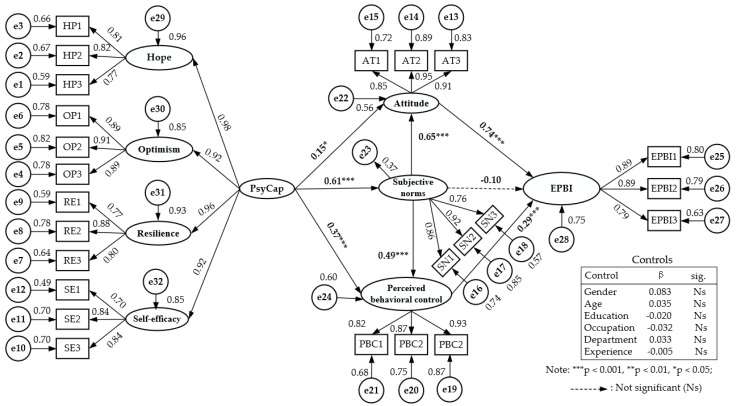
Structural equation model diagram of the influence of PsyCap on EPBI.

After testing, we found the absolute value of skewness of each observed variable was between 0.155 and 1.140, and the absolute value of kurtosis was between 0.022 and 3.520, so the data formed a normal distribution. The Chi-square versus Mahalanobis distance diagrams of variables were drawn by using the extension program “Normaltest_V1.0” of SPSS 26.0 software. The points in the map approximately formed a straight line, and the combination of all observed variables was close to multivariate normal distribution, so the maximum likelihood estimation method was used to estimate the model parameters. Considering that the overall fitting index value of the model is easily affected by the number of samples, this study selected χ^2^/df, RMR, RMSEA, and other indicators to verify the fit of the model and data. The results showed that all the indexes reached or approached the range of recommended standards (χ^2^/df = 2.677, RMR = 0.286, RMSEA = 0.083, TLI = 0.867, CFI = 0.880, IFI = 0.881), and there was a good fit between the model and the data. Figure 2 shows the structural model of this study.

### 4.3. Hypotheses Testing

Based on the premise that the above model fits well, we tested the hypothesis of this study, and Table 5 shows the specific results. Among them, MHO staff’s attitude towards emergency preparedness (β = 0.742, *p* < 0.001) and perceived behavioral control (β = 0.286, *p* < 0.001) significantly affected their EPBI, supporting Hypothesis 1 and Hypothesis 2. Furthermore, the subjective norms of MHO staff significantly affected their attitude (β = 0.648, *p* < 0.001) and perceived behavioral control (β = 0.494, *p* < 0.001), Hypotheses 4 and 5 were supported. Finally, the PsyCap of MHO staff had a significant influence on their attitude towards emergency preparedness behavior (β = 0.152, *p* = 0.022), perceived behavioral control (β = 0.367, *p* < 0.001), and subjective norm (β = 0.608, *p* < 0.001), Hypotheses 6–8 were supported. However, the subjective norms of MHO staff (β = −0.097, *p* = 0.087) did not significantly impact their EPBI, and Hypothesis 3 was not supported. A reasonable reason for this result may be bias in the study design. For example, there is no unified consensus on the definition of subjective norms in this study. However, this study mainly adopts mandatory subjective norms, whether the essential people around them support their EPBI, but does not consider the demonstrative subjective normative structure, such as how the important people around them do it themselves. Conversely, it may be because most human behaviors are under the control of self-will; thus, the EPBI of MHO staff is more affected by their attitude and perceived sense of behavioral control, making the role of subjective norms relatively weak.

In testing the mediating effect, we consider that the commonly used Sobel method has some limitations. Therefore, this study chooses the bootstrap method to test the mediating role of attitude, perceived behavioral control, and subjective norms in the three paths based on 5000 repeated samplings [57,58]. Table 6 lists specific verification results. Among them, the confidence interval of intermediary pat ① under the Bias-Corrected method at 95% confidence level is [0.005, 0.279], and the *p*-value is 0.040. The results show that the indirect effect is significant, and its estimated value is 0.122, supporting Hypothesis 9. The confidence interval of intermediary path ② under the Bias-Corrected method at 95% confidence level is [0.024, 0.262], and the *p*-value is 0.003. The results show that the indirect effect is significant, and its estimated value is 0.113, supporting Hypothesis 10. According to the same method of judgment, we found that paths ④ and ⑤ were also significant, supporting Hypotheses 12 and 13. However, the confidence interval of intermediary path ③ under the Bias-Corrected method at 95% confidence level is [−0.227, 0.076], and the *p*-value is 0.303. The results show that the indirect effect is not significant, Hypothesis 11 was not supported, echoing the fact that Hypothesis 3 is not valid in the hypothesis test.

## 5. Conclusions and Suggestions

With the continuous spread of global social risks, the emergency management system plays an increasingly important role in China’s national governance system [59]. However, improving the awareness of MHO staff’s emergency preparedness is a crucial way to enhance the foresight, scientificity, and initiative of emergency management. This study focused on the perspective of positive psychological movement and extended the theory of planned behavior by integrating PsyCap to investigate the mechanism by which EPBI is formed. Almost all the hypotheses were supported. The main results of this study are as follows: (1) Attitude and perceived behavioral control had significant positive effects on MHO staff’s EPBI, and subjective norms can positively influence attitude and perceived behavioral control. (2) Although subjective norms do not have a direct impact on EPBI, they will have an indirect impact through the intermediary roles of attitude and perceived behavioral control. (3) PsyCap had a significant influence on the decision-making process of MHO staff’s emergency preparedness behavior. Specifically, PsyCap had significant positive effects on attitude toward emergency preparedness behavior, subjective norms, and perceived behavioral control. Additionally, PsyCap affected MHO staff’s EPBI through the intermediary effects of attitude and perceived behavioral control.

### 5.1. Theoretical Contribution

Firstly, in the context of China, TPB can be used to predict and explain the intention of emergency preparedness behavior, which further expands the application field of TPB. Secondly, external motivation and internal opportunity work together to affect the EPBI of MHO staff. Most of the previous literature considered the influence of external environmental factors on the formation of EPBI, but seldom considered the influence of individual internal factors. However, the impact of external drivers on EPBI is not real-time and effective, which largely depends on individual self-management. Therefore, this study integrates the theoretical viewpoints in the field of positive psychology and organizational behavior, and further discusses the self-driving mechanism of MHO staff’s EPBI. Talking about the influence of PsyCap on EPBI enriches the existing research literature in the field of emergency preparedness, and its conclusions deepen our understanding of the formation mechanism of EPBI. Finally, this study complements the original TPB model by including role-width resources that can promote individuals to perform a broader range of tasks, which provides a more perfect theoretical model for predicting role-width behavior in MHO staff’s behavior database and provides an important update consideration for the development of a comprehensive behavioral model.

### 5.2. Practical Significance

Understanding the influence of TPB on EPBI and the influence of PsyCap on behavioral decision-making process are of great significance to the practical application of positive psychology and organizational behavior theory. Firstly, considering the impact of TPB mechanism on EPBI, management can take corresponding measures to encourage MHO staff to develop the necessary safety skills and knowledge, and stimulate their EPBI in multiple ways. These measures are as follows:

(1) Cultivate crisis awareness and improve the psychological risk reserve of MHO staff.
(2) Strengthen the training of emergency knowledge to make MHO staff fully aware of the significance and value of EP.
(3) Conduct emergency practice drills to enhance the confidence of MHO staff in dealing with unexpected accidents.
(4) Establish emergency logistics support work to ensure MHO workers’ health and life safety, etc.

Additionally, the influence of PsyCap on the behavioral decision-making process also provides a unique opportunity for managers to improve the enthusiasm of MHO staff to carry out emergency preparedness work. Managers can develop the PsyCap of MHO staff at the sub-structure or macro-level to improve their attitude towards emergency preparedness behavior, perceived behavioral control and subjective norms. The following are several measures to develop the PsyCap of MHO staff:

(1) Involve MHO staff in the process of preparing emergency preparedness and response plans.
(2) Make realistic and optimistic expectations to counteract the pessimism of MHO staff about emergency preparedness.
(3) Reinforce the transferable value of emergency preparation behavior in the career development of MHO staff.
(4) Provide positive feedback to MHO staff who are actively involved in emergency preparedness, etc.

### 5.3. Limitations and Prospect

This study has important theoretical and practical significance for understanding and stimulating MHO staff’s EPBI, but there are still some limitations. First, the subjects of this study are MHO staff, and the original data were collected through the form of a network questionnaire; hence, the homologous bias cannot be eliminated. Therefore, the research method should be further improved in subsequent studies. Second, the model in this study only considered the mediating effect and did not involve moderating variables. Therefore, future studies should consider more influencing variables to make the results more objective and comprehensive. Finally, the samples used in this study to verify the hypothesis are from China, and whether the research results can be inferred in other countries (regions) needs to be further verified. Follow-up research can increase the scope of the survey and sample size, so that the research results can adapt to a wider range of research objects, and improve the external validity of the research results.

## Figures and Tables

**Table 1:** Measurement items of latent variables.

Variables	Measurement Items
HP	1. If I should find myself in a jam, I could think of many ways to get out of it.
	2. At the present time, I am energetically pursuing my training goals.
	3. There are lots of ways around any problem.
OP	1. When things are uncertain for me at work, I usually expect the best.
	2. I’m optimistic about what will happen to me in the future as it pertains to work.
	3. I approach this job as if “every cloud has a silver lining.”
RES	1. When I have a setback at work, I have trouble recovering from it, moving on.
	2. I usually take stressful things at work in stride.
	3. I feel I can handle many things at a time at work.
SE	1. I feel confident analyzing a long-term problem to find a solution
	2. I feel confident in representing my work area in meetings with management.
	3. I feel confident contacting people outside my organization (e.g., patients) to discuss problems.
AT	1. I think it is important to participate in emergency preparedness.
	2. I think it is beneficial to participate in emergency preparedness.
	3. I think it is necessary to participate in emergency preparedness.
SN	1. My families encouraged me to participate in emergency preparedness.
	2. My friends encouraged me to participate in emergency preparedness.
	3. My managers encouraged me to participate in emergency preparedness.
PBC	1. I have enough skills of emergency preparedness.
	2. I have enough knowledge of emergency preparedness.
	3. I have sufficient resources for conduct emergency preparedness.
EPBI	1. I will actively participate in the emergency drills in response to major emergencies.
	2. I will actively participate in the preparation of public health emergency plans.
	3. I will actively popularize the knowledge and skills related to prevention of public health emergencies to the people around me.

Note: HP = hope; OP= optimistic; RES = resilience; SE = self-efficacy; AT = attitude; SN = subjective norms; PBC = perceived behavioral control; EPBI = emergency preparedness behavioral intention.

**Table 2:** Distribution of demographic characteristics of respondents.

Variables	Classification	Quantity	Percentage
Gender	Male	47	19.3
	Female	196	80.7
Age	18~24	91	37.4
	25~30	29	11.9
	31~40	70	28.8
	41~50	41	16.9
	51~60	12	4.9
Education	Senior high school degree or below	10	4.1
	College degree	58	23.9
	Bachelor degree	152	62.6
	Graduate degree or above	23	9.5
Occupation	Doctor	58	23.9
	Nurse	111	45.7
	The administrative staff of the hospital	12	4.9
	The professional staff of the CDC	1	0.4
	The administrative staff of the CDC	3	1.2
	The administrative staff of other health management departments	15	6.2
Department	Respiratory department	12	4.9
	Infection department	2	0.8
	Critical care department	2	0.8
	Otolaryngology Department	1	0.4
	Operating Room	5	2.1
	Emergency department	4	1.6
	Others	217	89.4
Experience	He/she had the experience of assisting Wuhan during the epidemic	99	40.7
	He/she had no experience of assisting Wuhan during the epidemic	144	59.3

**Table 3:** Analysis of reliability, content validity, and convergent validity of the scale.

Latent Variables	Observation Variables	Mean	SD	Estimate	CR	AVE	Cronbach’s α
HP	HP1	4.69	0.848	0.830	0.841	0.638	0.841
	HP2	4.74	0.859	0.807			
	HP3	4.86	0.753	0.758			
OP	OP1	4.71	0.891	0.891	0.921	0.796	0.921
	OP2	4.73	0.891	0.901			
	OP3	4.79	0.852	0.885			
RES	RE1	4.86	0.766	0.746	0.860	0.672	0.849
	RE2	4.51	0.981	0.888			
	RE3	4.47	1.017	0.820			
SE	SE1	4.74	0.819	0.697	0.834	0.628	0.835
	SE2	4.70	0.878	0.830			
	SE3	4.71	0.887	0.842			
AT	AT1	4.33	0.588	0.849	0.931	0.818	0.926
	AT2	4.36	0.589	0.948			
	AT3	4.36	0.610	0.913			
SN	SN1	4.04	0.751	0.859	0.884	0.719	0.874
	SN2	4.07	0.692	0.913			
	SN3	4.19	0.666	0.765			
PBC	PBC1	3.74	0.874	0.821	0.908	0.767	0.903
	PBC2	3.82	0.798	0.864			
	PBC3	3.66	0.877	0.939			
EPBI	EPBI1	4.18	0.674	0.884	0.891	0.732	0.887
	EPBI2	4.18	0.668	0.892			
	EPBI3	4.20	0.700	0.787			

**Table 4:** Analysis of discriminant validity of the scale.

Variables	HP	OP	RES	SE	AT	SN	PBC	EPBI
HP	0.799							
OP	0.873 ***	0.892						
RES	0.922 ***	0.923 ***	0.820					
SE	0.976 ***	0.814 ***	0.837 ***	0.792				
AT	0.535 ***	0.536 ***	0.445 ***	0.501 ***	0.904			
SN	0.586 ***	0.586 ***	0.562 ***	0.598 ***	0.747 ***	0.848		
PBC	0.578 ***	0.643 ***	0.662 ***	0.594 ***	0.532 ***	0.723 ***	0.876	
EPBI	0.635 ***	0.637 ***	0.570 ***	0.596 ***	0.821 ***	0.650 ***	0.606 ***	0.856

Note: *** *p* < 0.001; The diagonal of the matrix is the square root of the AVE, and below the diagonal is the correlation coefficient between variables.

**Table 5:** Path coefficient estimation and hypothesis test of the model.

Hypotheses	β Coefficient	S.E.	C.R.	*p*-Value	Is it Established?
Hypothesis 1:AT→EPBI	0.742	0.086	9.481	<0.001	Yes
Hypothesis 2:PBC→EPBI	0.286	0.054	3.934	<0.001	Yes
Hypothesis 3:SN→EPBI	−0.097	0.087	−1.051	0.293	No
Hypothesis 4:SN→AT	0.648	0.064	8.745	<0.001	Yes
Hypothesis 5:SN→PBC	0.494	0.086	7.291	<0.001	Yes
Hypothesis 6:PsyCap→AT	0.152	0.065	2.294	0.022	Yes
Hypothesis 7:PsyCap→PBC	0.367	0.099	5.377	<0.001	Yes
Hypothesis 8:PsyCap→SN	0.608	0.080	8.639	<0.001	Yes

**Table 6:** Test results of mediating effect of the model.

Paths	Indirect Effect	Bias-Corrected			Significance
		95%CI			
	Estimate	Lower	Upper	*p*-Value	
①PsyCap→AT→EPBI	0.122	0.005	0.279	0.040	Significant
②PsyCap→PBC→EPBI	0.113	0.024	0.262	0.003	Significant
③PsyCap→SN→EPBI	−0.063	−0.227	0.076	0.303	Not significant
④SN→AT→EPBI	0.452	0.310	0.703	<0.001	Significant
⑤SN→PBC→EPBI	0.133	0.055	0.250	0.002	Significant

Note: Lower= lower limit confidence interval; Upper = upper limit confidence interval.
